# Varicella infections in patients with end stage renal disease: a systematic review

**DOI:** 10.1186/s12882-018-0976-4

**Published:** 2018-07-24

**Authors:** Chong Yau Ong, Sher Guan Low, Farhad Fakhrudin Vasanwala, Shashidhar Baikunje, Lian Leng Low

**Affiliations:** 1 Department of Family Medicine, Sengkang General Hospital, 110 Sengkang East Way, Singapore, 544886 Singapore; 2 Post-acute and Continuing Care, SingHealth Community Hospital (Sengkang), Singapore, Singapore; 3 SingHealth Duke-NUS Family Medicine Academic Care Program, Singapore, Singapore; 4 Department of General Medicine, Sengkang General Hospital, Singapore, Singapore; 5 Department of Renal Medicine, Singapore General Hospital, Singapore, Singapore; 6 Department of Family Medicine and Continuing Care, Singapore General Hospital, Singapore, Singapore

**Keywords:** Varicella, Chickenpox, End stage renal failure, End stage renal disease, Varicella vaccine, Impact, Morbidity, Mortality

## Abstract

**Background:**

End stage renal disease (ESRD) is on the rise globally. Varicella infection among adult patients with ESRD has been reported to lead to multiple complications and even death. While varicella vaccination has been recommended in paediatric renal patients; recommendation on varicella vaccination among adult patients with ESRD remained sparse. This review is aimed at evaluating the impact of varicella infection among adult patients with ESRD and make a recommendation for vaccination.

**Methods:**

Three databases (PubMed, Embase and Cumulative Index to Nursing and Allied Health Literature (CINAHL)) were searched in April 2018 with keywords ‘varicella, chronic kidney failure, chronic kidney disease, renal replacement therapy, kidney transplantation, end stage renal disease, end stage renal failure, chicken pox, vaccine, vaccination and complications’.

**Results:**

29 articles were selected for review. The studies were mainly case reports, and they included measured outcomes: prevalence of seronegativity, impact (morbidity, length of stay, and mortality) of varicella among patients with ESRD, seroconversion rates and safety of varicella vaccination. The prevalence of seronegativity among varicella-infected ESRD adults was found to be at 42 to 100%. Nineteen deaths were reported. At least 54 patients have had complications from varicella infection. Seroconversion rate post vaccination was found to be around 64–94%.

**Conclusion:**

Varicella is associated with significant morbidity and mortality rates in adult patients with ESRD. Varicella vaccination should be considered for the vulnerable, seronegative patients.

## Background

End stage renal disease (ESRD) is a prevalent chronic condition in many countries. ESRD incident rate in developed countries had largely stabilized in the past one decade, although incident rates rose for many developing countries during the same period [1]. The lifetime risk for an individual to develop chronic kidney disease (CKD) is high, with more than half the adults aged 30–64 years in the United States likely to develop CKD [2]. About 2.6 million people were on dialysis in 2010; 93% in high or upper-middle-income countries [3]. By 2030, worldwide use of renal replacement therapy (RRT) is projected to more than double, with a most projected increase in Asia [3].

Patients with ESRD have impaired immune system and therefore are susceptible to infections [4]. The disturbance to the immunity system is caused by uraemia, haemodialysis procedure, complications of CKD and therapeutic interventions for their treatment. Fehr et al.’s literature review on cases of disseminated varicella infection in adult renal allograft recipients, showed an overall mortality of 34% [5]. The mortality rate from pulmonary infections was 14 to 16-fold higher in dialysis patients and about two-fold higher in renal transplant recipients compared to general population [6]. One large cohort observational study showed hazard ratio of hospitalisation due to infection among patients with CKD or ESRD to be as high as 2.55 with a corresponding hazard ratio of 3.76 for infection-related deaths [7].

Varicella (chickenpox) is a primary infectious disease that is caused by varicella-zoster virus (VZV), an alpha herpes virus belonging to the Herpesviridae family. The secondary household attack rate of over 90% showed that varicella is highly contagious [8]. Transmissions are mostly airborne and by direct contact with vesicular fluids. The course of the disease is usually benign among paediatric patients; however, this is not so with adult patients. When it occurs in adult renal transplant recipients, it follows a virulent course and carries a very high risk of morbidity and mortality [9, 10]. Pneumonia, pneumonitis, acute obstructive respiratory disease, encephalitis, meningitis, neutropenia, thrombocytopenia, Henoch-Schonlein purpura, synovitis, Reye’s syndrome, secondary bacterial infections (sepsis, cellulitis, impetigo, abscesses, necrotizing fasciitis, and toxic skin syndrome) - the list of possible complications from varicella infection are numerous.

Since the advent of varicella vaccination, it had been proven to be effective in seroconverting paediatrics patients (including children with leukaemia), adolescents and adults, with a low occurrence of vaccine-associated rash among immunocompetent patients [11]. Similarly, seroconversion rates in adults have been encouraging, although adults respond less effectively than children group. In adults with ESRD, there are few studies on the efficacy of varicella vaccination in seroconverting this group of patients who are known to respond less efficiently to vaccinations. This is followed by lack of consensus and guidelines recommendation on vaccinating ESRD patients with VZV vaccines. This review is aimed at identifying the prevalence of seronegativity among patients with ESRD, evaluating the impact of varicella infection to adult patients with ESRD, and synthesizing current recommendations on VZV vaccination.

## Methods

### Data sources and search terms

The relevant papers published were collected through a computerised search on three databases (PubMed, Embase and Cumulative Index to Nursing and Allied Health Literature, CINAHL) using the keywords: chronic kidney failure, renal replacement therapy, kidney transplantation, end stage renal disease, end stage renal failure, chicken pox, varicella, vaccine, vaccination and complication. For PubMed search, the Boolean search of (Kidney Failure, Chronic [Medical Subject Heading (MeSH) Terms]) OR Renal Replacement Therapy [MeSH Terms]) OR kidney transplantation [MeSH Terms]) OR end stage renal disease) OR end stage renal failure)) AND (“Chickenpox”[MeSH Terms]) OR “Varicella”) AND (Complicat* OR vaccin*) was used. The same search terms were used for Embase and CINAHL database searches. For CINAHL only academic journals were included, periodics and bulletins were not included. The search was conducted in April 2018. There was no time frame limitation applied for the inclusion of the studies.

### Study selection and eligibility criteria

Two reviewers, O.C.Y and L.S.G, independently evaluated the articles for eligibility through screening of the title and abstract first, followed by full text. Consensus on the eligibility of the articles was sought, and F.F.V was involved if there was disagreement and would act as an adjudicator.

A study is included if it is found to be relevant with regards to varicella infection in ESRD: the prevalence of seronegativity, the complications of the infection, or safety and efficacy of varicella vaccination to adult patients with ESRD or CKD. Case reports and cohort were included if measurable outcomes of death, complications, or length of stay were described. Records on herpes zoster, acyclovir, and non-renal solid organ transplants were excluded. Records on paediatric/ child populations were excluded.

### Data analysis

Selected studies were summarised in Table 1. The data was grouped into themes of seroprevalance, impact of the disease, immunogenicity and safety of the varicella vaccination. Each article was graded for quality of study based on the Strength of Recommendation Taxonomy (SORT); which was introduced by the United States family medicine and primary care journals (i.e., American Family Physician, Family Medicine, The Journal of Family Practice, Journal of the American Board of Family Practice, and British Medical Journal-USA) and the Family Practice Inquiries Network (FPIN) [12]. The SORT was used because it can be applied to many sources of evidence and therefore suitable for our review which included studies with heterogeneous designs. Study quality was included in Tables 2, 3, 4, and 5. Risks of bias of each study were not accessed directly as most studies were of grade three in qualities based on the SORT. No statistical analysis was performed.

**Table 1:** Characteristics of selected studies

Study	Region	Design	Study population	Outcomes measured			
				Prevalence of disease/immunity	Morbidity/ Mortality	Efficacy	Safety
Crespo JF, et al. (2002) [16]	Spain	Prospective cohort	Single centre. 336 candidates for renal transplant. Follow-up 4 years.	+		+	+
Geel AL, et al. (2006) [17]	Netherlands	Prospective cohort	Single centre. 854 transplants patients. 286 waitlist patients. Follow-up 13 weeks.	+		+	+
Rodríguez-Moreno A, et al. (2006) [13]	Spain	Retrospective data collection	Single centre. 812 adult renal transplant patients. (From 1995 to 2004).	+	+		
Kaul A, et al. (2012) [9]	India	Retrospective data collection	Single centre. 1546 adult renal transplants patients. (From June2000-June 2010)	+	+		
Talebi-Taher M, et al. (2013) [18]	Iran	Cross sectional	Single centre. VZV IgG acquisition from 187 haemodialysis patients (aged 18 to 88). (March–July 2010).	+			
Abad CL, et al. (2016) [14]	USA	Retrospective data collection	Not available. Review of all cases with disseminated VZV among renal transplant recipients 56 cases in adults.(From 1985 to 2011).	+			
Ong CY, et al. (2018) [15]	Singapore	Retrospective data collection	Single centre. Review of all cases with varicella among ESRD patients. 66 cases in adults. (From 2005 to 2016).	+	+		
Errasti P, et al. (1999) [19]	USA	Case reports from retrospective data collection	Single centre. Review of 476 renal transplant recipients revealed 4 cases of chickenpox. (Renal transplant done from 1969 to 1998).		+		
Ishikawa N, et al. (2000) [20]	Japan	Case reports	2 patients described.		+		
Fehr T, et al. (2002) [5]	i)not mentioned ii) Switzerland	i) Review of literature. ii)Case reports	i) Not available. Review of literature 1981–2000. 34 cases disseminated varicella identified. ii) 4 cases reported.		+		
Lauzurica R, et al. (2003) [21]	USA	Retrospective data collection	Single centre. Review of kidney transplant recipients.1 patient described. (Oct 1985 to Aug 2002).		+		
Sinha S, et al. (2003) [46]	India	Case reports	2 patients described.		+		
Robertson S, et al. (2006) [22]	Scotland, UK	Case report	1 patient described.		+		
Shahabazian H, et al. (2007) [47]	Iran	Case report	Report of chickenpox outbreak in renal transplant recipients. 3 patients described.		+		
Crowther N, et al. (2009) [31]	Australia	i) Retrospective data collection. ii) Case report	Single centre. Review of renal allograft recipients revealed 1 patient developed varicella. (From Dec 1972 to July 2010)		+		
Kandasamy R, et al. (2009) [48]	USA	Case report	1 patient described.		+		
Sato A, et al. (2009) [27]	Japan	Case report	1 patient described.		+		
Assi M, et al. (2011) [29]	USA	Case report	1 patient described.		+		
Mustapic Z, et al. (2011) [49]	Croatia	Case report	2 patients described.		+		
Chiang E, et al. (2012) [50]	USA	Case report	1 patient described.		+		
Inokuchi R, et al. (2013) [23]	Japan	Case report	1 patient described.		+		
Low LL, et al. (2014) [30]	Singapore	Case report	1 patient described.		+		
Nabi S, et al. (2014) [26]	USA	Case report	1 patient described.		+		
Sampathkumar K, et al. (2015) [24]	India	Case report	1 patient described.		+		
Depledge DP, et al. (2016) [25]	UK	Case report	1 patient described.		+		
Chhabra P, et al. (2017) [51]	India	Case report	1 patient described		+		
Momani H, et al. (2017) [52]	Jordan	Retrospective data collection.	Single centre. 20 renal transplants patients revealed 1 patient developed varicella. (From April 2015–June 2016)		+		
Kho MML, et al. (2017) [32]	Netherlands	Prospective cohort	Not available. 52 kidney transplants patients. Follow-up two years.			+	+
Scanlon-Kohlroser CA, et al. (2002) [28]	USA	Case report	1 patient described.				+

+Outcomes measures available

**Table 2:** Prevalence of seronegative results

Reference	Main Results			Timing of serology taken	Main conclusions	Study quality
	Renal transplant patients/recipients	Haemodialysis patients	Renal transplant candidates +			
Crespo JF, et al. (2002) [16]			Among 336 renal transplant candidates, 33 (9.8%) were seronegative.	Before contraction of primary varicella	–	Level 2
Geel AL, et al. (2006) [17]	Among 854 transplant recipients, 2.1% were seronegative.		Among 286 patients on the wait list, 3.2% patients were seronegative	Before contraction of primary varicella	-Low prevalence of seronegativity. -At risk of severe complications after contact with chickenpox.	Level 2
Rodríguez-Moreno A, et al. (2006) [13]	Among the four patients that developed primary varicella infection, all were tested negative for VZV IgG.			Presentation/onset of primary varicella	-Varicella infection among renal allograft recipients is unusual but carries a high morbidity and mortality.	Level 3
Kaul A, et al. (2012) [9]	Among 23 renal allograft patients that developed varicella infection, all was tested negative for VZV IgG.			Presentation/onset of primary varicella	–	Level 3
Talebi-Taher M, et al. (2013) [18]		Among 187 patients on haemodialysis, 2.1% were seronegative.		Before contraction of primary varicella	-No correlation between patient’s self-reported history of VZV infection and seroprevalence status (*p* = 0.6). -Serologic screening for VZV for transplant candidates is essential. -Consider this population as a target group for future national immunisation program.	Level 2
Abad CL, et al. (2016) [14]	Among 54 cases of varicella in transplant recipients, baseline serology available in 32 patients, 19 (59.4%) were seronegative.			Presentation/onset of primary varicella	Baseline serologies before transplantation remains useful as markers for prior exposure and latent infection. It also guides VZV vaccination.	Level 3
Ong CY, et al. (2018) [15]	Among 66 cases of varicella in patients with ESRD (dialysis, transplant, conservative), baseline serology available in 19 patients. 42.1% were seronegative.			Presentation/onset of primary varicella	-Immunity to varicella should be screened among ESRD patients. -Seronegative patients to be considered for varicella vaccination.	Level 3

+ Information on whether renal replacement or no renal replacement therapy given while awaiting transplant were not mentioned

**Table 3:** Impact of the disease: mortality and morbidity

Reference	Patient’s presentation	Results			Elaborations on results	Main conclusions	Study quality
		Complication	Length of stay (LOS)	Mortality			
Ong CY, et al. (2018) [15]	-66 patients developed varicella in the 12-year review of all ESRD patients. -Age range: 19–89 years old (median:53) -37 male patients. -Timing of infection: 6 to 19 years post diagnosis of ESRD.	+	+	+	-24 patients developed at least one complication. Encephalitis, meningitis, pneumonia/pneumonitis. -LOS: median 10 days -9 died (13.6%)	-ESRD patients had significant morbidity and mortality associated with varicella infection. -Screen for seronegative patients and consider vaccinate them.	Level 3
Errasti P, et al. (1999) [19]	-31 y.o. Woman, 5 years post-transplant, admitted for acute epigastric pain with 3 days vesicular rash.	+	NA	+	-Multiorgan failure: -Fulminant hepatitis (post-mortem showed massive hepatic necrosis). -Died in 2 days.	-Chickenpox often follows severe and often fatal course in adults with renal transplantation. -Vaccine appears to prevent clinical varicella following subsequent exposure.	Level 3
	-29 y.o. Man, 17 years post-transplant, admitted for confluent-haemorrhagic rash.	+	NA	+	-Encephalitis (post-mortem showed cerebral oedema). -Disseminated intravascular coagulation (DIC) with multiple bleeding sites. -Multiorgan failure. -Secondary Staphylococcus bacteraemia. -Patient died.		
	−59 y.o. Man, 2 years post-transplant, had few vesicular rash. Exposed to his son who had varicella 4 weeks ago.	–	NA	–	-No complication		
	-69 y.o. Woman, 8 months post-transplant, admitted for vesicular rash and fever.	–	NA	–	-No complication		
Ishikawa N, et al. (2000) [20]	-29 y.o. Man, 11 months post-renal transplantation. With papular and vesicular rash and abdominal pain.	+	NA	–	-DIC and gastrointestinal bleeding.	-Varicella vaccination should be administered before transplantation if patients had no past varicella infection based on history and antibody titre	Level 3
	-36 y.o. Woman with a vesicular rash on face. Had renal transplant 3 years ago.	+	NA	–	-DIC		
Fehr T, et al. (2002) [5]	-51 y.o. man, 11 years post-transplantation, had abdominal pain, nausea, vomiting, and generalised pustulosis.	+	NA	–	-Pneumonitis and hypoxic respiratory failure. -Failure of graft 6 months later.	-Overall mortality of 34%. Mortality after 1990 with acyclovir and reduction of immunosuppressants were 22%. −82% of patients summarised had substantial mortality. -Vaccination is effective and has no severe side effects. -Routine VZV serology test for every ESRD patients before renal transplant. -Vaccination in those with negative or very low VZV antibody titres.	Level 3
	-34 y.o. Man, 1.5 years post-transplant, had acute epigastric pain, nausea, vomiting, and vesicular rash.	+	NA	–	-DIC, hepatitis.		
	-51 y.o. Man, 6 months post-transplant, admitted for progressive dyspnoea.	+	+	–	-Pneumonitis with respiratory failure. -LOS: 26 days.		
	-23 y.o. Man, 6 months post-transplant, presented with vesicles whole body.	+	+	–	-Hepatitis -LOS: 10 days		
Lauzurica R, et al. (2003) [21]	-30 y.o. Man presented with vesicular-pustular rash, fever and abdominal pain, 3.5 years post-transplant.	+	NA	+	-Pneumonitis with respiratory failure -Mild transaminitis. -Died 4 days upon admission due to multiorgan failure: (hepatitis, myocarditis, DIC)	-Detecting VZV seronegative patients before the renal transplant is relevant because vaccination may minimise the risks of future infection.	Level 3
Sinha S, et al. (2003) [46]	-22 y.o. Woman, 42 months post-transplant, presented with abdominal pain 1 week after the development of chickenpox.	+	NA	–	-Pancreatitis.	-Acute pancreatitis as a consequent of viral infection is well known	Level 3
	-36 y.o. Man, 10 days post-transplant, developed pancreatitis 2 weeks after pancreatitis.	+	NA	–	-Mild acute pancreatitis		
Robertson S, et al. (2005) [22]	-30 y.o. Man with a generalised maculopapular rash	+	NA	+	-Fulminant varicella with multiorgan involvement (acute renal failure, acute liver failure) - Died within 60 h of admission	-Although regarded mild infection in children, chickenpox can cause fatality in adults and in the immunocompromised. -Screen potential renal transplant recipients for VZV susceptibility and offer vaccination to the seronegative patients. -Test for immunity for varicella as soon as progressive renal failure is diagnosed.	Level 3
Rodríguez-Moreno A, et al. (2006) [13]	-Eight patients (1%) developed varicella (7 men, 1 women). -Age range: 32–64. -Median time from transplantation to infection was 32mths.	+	+	+	Complications: - 2 pneumonitis, 1 hepatitis, 1 thrombotic microangiopathy, 1 multiorgan failure - LOS: 11 days (median 3 to 21). - One (12.5%) death due to multiorgan failure	-Varicella infection in adult allograft recipients is unusual but highly morbid -Vaccination of seronegative pre-transplant candidates should be attempted	Level 3
Shahbazian H, et al. (2007) [47]	-37 y.o. Man, a year post-transplant, admitted for severe abdominal pain.	+	+	–	-Acute kidney injury -LOS: 10 days	-All renal transplant recipients should be screened for VZV immunity before transplant irrespective of previous VZV infection. - Seronegative patients should receive live VZV vaccine several months prior to transplant.	Level 3
	-44 y.o. Man, 9 years post-transplantation, admitted for low back pain of 2 days duration. 2 days later he developed fever and papulovesicular rash 2 days later	–	+	–	-LOS: 15 days		
	-34yo man, 8 years post-transplantation, admitted for acute abdominal pain with intractable nausea vomiting. Papulovesicular rash appeared on the face and trunk 48 h later before became generalised.	–	+	–	-LOS: 13 days		
Crowther N, et al. (2008) [31]	-43 y.o. Man, 16 years post-renal transplant. Acute renal failure detected at routine clinic review. He had scattered skin lesion after his children had chickenpox 2 weeks ago.	+	NA	–	-Diagnosis: late acute mediated rejection post-transplant precipitated by recurrent varicella infection.	–	Level 3
Kandasamy R et al. (2009) [48]	-58 y.o. Man with fever and progressive rash	+	NA	–	-Darrier disease related to disseminated varicella	–	Level 3
Sato A, et al. (2009) [27]	-36 y.o. Woman presented with an irritable cough	+	+	–	-Varicella pneumonia -LOS: 1 month and 10 days	-One should keep the possibility of VZV reinfection in mind, in Immunocompromised patients with past history of varicella.	Level 3
Assi M, et al. (2011) [29]	-68 y.o. man with kidney transplant 10 years ago, presented with 5-days fever, confusion and altered sensorium	+	NA	–	Varicella encephalitis, followed by Guillain-Barre syndrome (GBS).	–	Level 3
Mustapic Z, et al. (2011) [49]	-Two renal allograft patients developed varicella. Details unavailable.	NA	NA	NA	-Not available	-VZV infection is a rare but potentially serious complication in renal transplant recipients. -Active immunisation for VZV-seronegative patients before transplantation should be performed.	Level 3
Chiang E, et al. (2012) [50]	-42 y.o. Woman, unknown years post kidney transplant, had right eye redness, tearing, and blurred vision for 1 month.	+	NA	–	-Acute retinal necrosis	–	Level 3
Kaul A, et al. (2012) [9]	-23 patients developed varicella in the 10-year review of post renal transplant. -Age range: 21–54 years old (median:39) -17 male patients. -Timing of infection: < 15 days post-transplant to > 5 years post-transplant.	+	NA	+	-5 had graft dysfunction. - 7 had infections (6 bacterial, 1 fungal). - 3 had sepsis - 5 had gastritis - 2 had encephalitis - 2 had pancreatitis - 2 had orchitis - 2 died (8.6%)	-Primary varicella/chickenpox is a potentially fatal infection in adult renal transplant recipients. -Varicella vaccination in the high-risk groups, especially during the pre-ESRD stage, may reduce the number of varicella infection.	Level 3
Inokuchi R, et al. (2013) [23]	-A 69 y.o. Woman (20 years ESRD on dialysis, then 1 month post renal transplantation) presented with generalised rash one day.	+	NA	+	-Varicella pneumonia with respiratory failure. -Demised at Day 28 illness (despite change of antiviral to foscarnet on day10, mechanical ventilation on day3)	-Patients with VZV pneumonia with deep and vast ulcerations on bronchoscopy had fatal outcomes.	Level 3
Low LL, et al. (2014) [30]	-58 y.o. Man on haemodialysis, presented with fever and cough. Subsequently developed a papulovesicular rash on the 4th day of admission.	+	NA	–	-Varicella pneumonia -Varicella encephalitis	-Renal Physicians and Family Physicians in the Asia-Pacific region should study the epidemiological data in each country. -Consensus guidelines needed and how the varicella vaccination program can be targeted for those at risk. -Live attenuated varicella vaccine is has been proven to be safe when administered to adult ESRD patients regardless of RRT mode.	Level 3
Nabi S, et al. (2014) [26]	-73 y.o. Woman with kidney transplantation and recent CMV infection, presented with altered mental status.	+	NA	–	-Varicella encephalitis	-Disseminated VZV with encephalitis is rare, but a life-threatening condition	Level 3
Sampathkumar K, et al. (2015) [24]	-34 y.o. Man had kidney transplant 10 months ago, came with fever × 2 weeks and bitemporal headache.	+	NA	–	-VZV induced central nervous system angiopathy	–	Level 3
Depledge D, et al. (2016) [25]	-55y.o. Man post renal transplant day23 presented with abdominal pain, macular rash and abnormal liver function test.	+	NA	–	-VZV pneumonitis, hepatitis	-Risk of airborne transmission of VZV is evident, especially when viral load is high. - Immunocompromised patients are vulnerable to serious infection. - Need for pre-transplant vaccination.	Level 3
	-61y.o. Man post renal transplant day25 presented with 4 days fever, vesicular rash and abnormal liver function.	+	NA	+	-VZV hepatitis. - Died on day 6 admission (3 days in ICU)		
Chhabra P, et al. (2017) [51]	-33y.o. Man, 3 years post-transplant, had severe epigastric pain for 7 days.	+	NA	–	-Varicella pancreatitis and hepatitis	–	Level 3
Momani H, et al. (2017) [52]	-One patient developed varicella -Details unavailable	+	NA	–	-Varicella pneumonitis	–	Level 3

*NA* Not available

**Table 4:** Immunogenicity of varicella vaccination

Reference	Number of patients studied	Number of dose of VZV vaccine	Seroconversion rate/response rate	Main conclusions	Study quality
Crespo JF, et al. (2002) [16]	17	2	-94.1% after second dose of VZV vaccination.	-Vaccination protocol is effective in seroconverting.	Level 2
Geel AL, et al. (2006) [17]	11	2	-64% seroconverted after two doses of VZV vaccine.	-64% seroconversion was lesser than post-licensure studies. -Impaired immune system was responsible for less ability to mount antibody titres and maintaining it over time.	Level 2
Kho MM, et al. (2016) [32]	52	2	-40 responders (77%) found (AUC > 0.9) VZV specific antibody (Ab) at 3 months. -At one year, 67% still have positive VZV Ab. -At two years,45.8% have positive VZV Ab	-Two-dose vaccination before kidney transplantation regime is safe and effective in adults with CKD, resulting at least 77% seroconversion in VZV IgG and VZV-specific T cell memory.	Level 2

**Table 5:** Safety on varicella vaccination

Reference	No of patient studied	Complications of vaccine	Main conclusions	Study quality
Crespo JF, et al. (2002) [16]	-17 seronegative patients completed vaccination protocol.	-No secondary effect of vaccination detected. -None of the subsequently seroconverted patients who received kidney transplant presented with VZV disease (up to 18 months post renal transplant).	-Systematic vaccination prior to transplantation could prevent severe varicella.	Level 2
Scanlon-Kohlroser CA,et al. (2002) [28]	-A single case of 51yo woman at 6 months post-renal transplant developed a mild rash. -She had daily household contact with 15-month old twins vaccinated 40 days ago.	-Characteristic popular and vesicular rash over the face, trunk, extremities. No dissemination. Confirmed with positive VZV IgG 2 weeks later.	-Transmission from those vaccinated to susceptible individuals are rare and typically occurs only if these patients develop a rash. - Contact cases develop a subclinical infection or mild illness; suggesting vaccine virus remains attenuated when vaccinated.	Level 3
Geel AL, et al. (2006) [17]	-11 seronegative patients have been vaccinated with two doses VZV vaccine.	- No side effects, no fever, or skin lesions among all vaccinated patients.	-Vaccination should be performed in this group of patients in view of potentially lethal complications of primary varicella infection.	Level 2
Kho MML, et al. (2016) [32]	-52 seronegative patients given two doses of VZV vaccine.	-No severe vaccine-related adverse events were reported. - One had pain at injection site. -Two had zoster (3 months and 9 years post vaccination) -One patient developed mild varicella (18 days post vaccination).		Level 2

## Results

**Fig. 1:**
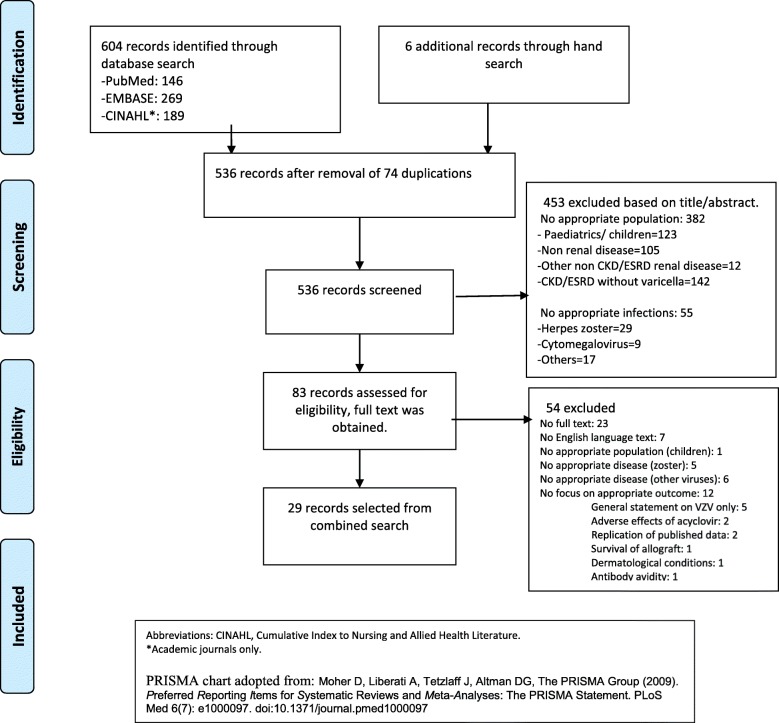
Details of article selection process in the literature search

610 studies were retrieved from the search strategy. After removal of duplications, 536 records remained. Screening of title and abstract narrowed down the number of records to 83 which were then assessed for eligibility. Twenty-nine studies were included in this review after study selection process (Fig. 1). More than half of the studies were case reports; the remaining studies comprised of retrospective data collection, prospective cohort, and cross-sectional studies (Table 1).

### Prevalence of varicella seronegativity among patients with ESRD

Out of the seven studies on the prevalence of seronegative results; four studies were on the prevalence of seronegativity among ESRD patients upon presentation of the varicella disease [9, 13–15]. The results showed that 42 to 100% of the patients who contracted varicella had no prior immunity to varicella. Three studies examined the prevalence of seronegativity among ESRD patients before contraction of primary varicella. Of the three, the first studied on transplant recipients [16], the second on both transplant recipients and candidates on waitlist [17], and the third on haemodialysis patients [18]. The latter three studies, however, showed that prevalence of seronegativity was low (2.1 to 9.8%).

The prevalence of VZV seronegativity varies among renal transplant recipients, haemodialysis patients, and renal transplant candidates awaiting transplant (Table 2). There was no mention of whether the candidates waiting transplant was on renal replacement therapy or not. Among transplant patients (*n* = 935), there was a huge range of prevalence seronegativity from 2.1 to 100% [9, 13, 14, 17]. Among haemodialysis patients (*n* = 187), the prevalence of seronegativity was 2.1% [18]. As for candidates awaiting transplant (*n* = 622), 3.2 to 9.8% was seronegative to VZV [16, 17].

### Impact of the disease (mortality and morbidity)

23 articles reported on the impact of the disease; including complications from varicella, length of stay, and mortality (Table 3). Collectively, there were nineteen deaths reported from the studies. Errasti, et al. reported four patients in which two died; both patients had significant complications (one with fulminant hepatitis, one had encephalitis) and multiorgan failure [19]. On the other hand, two other patients that had no complications survived the infection. Ishikawa, et al. reported two patients with disseminated intravascular coagulation [20]. Fehr et al. reported four cases in which all survived while their review of the literature revealed overall varicella mortality rates to be 34% [5]. Other deaths from varicella in ESRD were due to respiratory failures (one from pneumonia, one from pneumonitis), multiorgan failure (two cases), nervous system neuropathy (one case) and hepatitis (one case) [13, 21–25]. Length of stay has been reported to vary from 2 to 40 days. Other reported complications were pancreatitis, retinal necrosis, secondary bacterial infection, acute kidney injury, myocarditis, microangiopathy, Darrier’s disease, and even Guillain-Barre syndrome.

Most of the studies revealed that infected with primary varicella were treated with intravenous acyclovir. Standard dose of 10 mg/kg 8hourly (eight to fourteen days) were described in most cases (12 studies), renal adjusted dose were mentioned in seven reports, no dose of intravenous acyclovir was given in two reports, and in one study [9], all patients were given regimen of two weeks of intravenous acyclovir followed by three months of oral acyclovir was administered. One case was treated with three months of oral acyclovir. One case was treated with intravenous valaciclovir [26]. Intravenous ganciclovir was given in two cases [5, 9]. Cessation and reduction of immunosuppressant d rugs were described in four cases [5, 21, 25, 27, 28] and two studies [5, 9] respectively. Adjunctive antibiotics were initiated in five cases [5, 25, 27, 29, 30]. Foscarnet was given in one case following failure of initial treatment [23]. Immunoglobulins were administered in eight cases [13, 20, 31].

### Immunogenicity and safety of varicella vaccination

Three studies examined the seroconversion rate or post vaccination after administration of two doses of varicella vaccine. All three studies have limited number of patients. Crespo, et al. [16] reported a highly encouraging response rate of 94% while Geel, et al. [17] and Kho, et al. [32] found that the response rate to be around 64–77%. Table 4 summarises the seroconversion rates of selected studies.

As far as safety is concerned, Crespo, et al. and Geel, et al. found no secondary effect of vaccination [16, 32]. None of their vaccinated patients developed the varicella-zoster disease. Kho, et al. followed up 52 patients post-vaccination for complications and found one to have primary varicella and two to have herpes zoster [32]. Only one reported pain at injection site, no cellulitis or skin infection was reported. Interestingly, Scanlon-Kohlroser, et al. reported a case where transmission of varicella took place from two infants that were vaccinated to a post-renal transplant patient [28]. Table 5 summarises the complications of the vaccine.

## Discussion

### Summary of findings

In this review, the prevalence of seronegativity among varicella-infected ESRD adults was found to be significantly alarming at 42 to 100% [9, 13–15]. Nineteen deaths were reported in 23 studies that reported the varicella infections. At least 52 patients were reported to have complications from varicella infections. Efficacy of vaccination (measured by seroconversion rate after two doses of VZV vaccine) was found to be around 64–74%. Safety of vaccines showed that adverse effects or complications from vaccinations were zero in a cohort of fewer than twenty persons [16, 17]. Four adverse effects from vaccinations were reported in a study of 52 patients [32].

Varicella has been recognised as a potentially fatal disease among adults even though it has been largely regarded as a benign disease of childhood [33]. Although accounting for only 5% of reported cases of varicella, adults in general population contributed to 35% of all varicella deaths [34]. Furthermore, varicella is a more severe threat to adult patients with ESRD the myriad of organ and system-complications described. This dismisses the general perception of acute varicella being a self-limiting disease.

In the general population (adults and paediatrics), mortality rates were around 0.41 deaths per 1 million through 1990–1994. This decreased drastically to 0.14 deaths per 1 million during 1999 through 2001 [35, 36]. Compared to general population, mortality rates of varicella among adult patients with ESRD is much higher; suggesting the vulnerability of this group of patients to varicella infection.

Varicella-related complications derived from the review were no different from known complications of varicella infection [34]. Pneumonia, hepatitis, and encephalitis were found to be the leading complications. These complications may progress to multi-organ failure with high mortality.

Based on this review, seroconversion rates of 64–94% are encouraging and reflecting high immunogenicity when administered. This is in keeping with findings of live-attenuated varicella vaccinations being immunogenic, efficacious and safe in preventing varicella infections [35, 37]. Besides that, there are no major adverse effects in the cohort studies of vaccinated adult patients. This could suggest the positive role of vaccinating VZV seronegative patients with ESRD in preventing varicella infection.

In addition to the database search, we also searched specifically for guidelines on varicella vaccinations. As for recommendations for varicella vaccination in this group of patients; only a handful recommendations from published guidelines were found. The Advisory Committee on Immunization Practices (ACIP); Centres for Disease Control and Prevention (CDC) have recommended varicella vaccine for ESRD patients, who meet age criteria and who do not have contraindications to vaccine [38].

The American Society of Transplantation and the American Society of Transplant Surgeons recommended pre-transplantation VZV serology checking. Seronegative adults should receive one dose of varicella vaccine with serologic testing post vaccination. If seroconversion does not occur, the dose may be repeated once if time permits [39].

Similarly, the Korean Vaccination Society has recommended varicella vaccination for the seronegative adults; and this should be completed at least one month before transplantation [40]. The 2013 Infectious Disease Society of America (IDSA) Clinical Practice Guideline (CPG) for vaccination of the immunocompromised host advocated that varicella vaccine (VAR) should be given to immunocompetent patients without evidence of varicella immunity if it can be administered at least four weeks before initiating immunosuppressive therapy [41].

Both the US Department of Veterans Affairs and Department of Defence (2014) on their Clinical Practice Guideline for the Management of Chronic Kidney Disease in Primary Care (strong recommendation); and Public Health Agency of Canada (in their Canadian Immunisation Guide 2016) have extended the recommendation to include patients with chronic kidney disease or chronic renal disease [42, 43]. The Kidney Disease: Improving Global Outcomes (KDIGO) and the National Kidney Foundation’s Kidney Disease Outcome: Quality Improvement (KDOQI) have not specifically advocated for varicella vaccination post-transplant, the reason being varicella vaccine is a live-attenuated vaccine [44, 45]. At present, there is yet to be any recommendation by both KDIGO and KDOQI on pre-transplant vaccinations in general. While post-exposure prophylaxis with varicella immunoglobulin, and primary varicella treatment with acyclovir or valaciclovir has been recommended; they are still silent with regards to VZV immunisation as a preventive method [43, 45].

### Clinical implications

There is a lack of guidelines in the Asia Pacific Region on varicella vaccination in patients with ESRD. Since most patients with ESRD or advanced CKD are managed by renal physicians and family physicians; it is critical to advocate, initiate planning, followed by implementing policies on varicella vaccination among these susceptible patients. This is of increasing importance considering the increasing number of patients developing ESRD in Asia.

### Limitations and future research

The first limitation is the heterogeneity of the population in the studies that were included. The aim of this review is to review the available literature of adult populations with ESRD comprehensively. However, most studies included only subset populations of ESRD; namely renal transplant recipients or patients on haemodialysis and therefore findings may not be fully representative of the overall population of ESRD. Therefore, there is a real need for study varicella among patients with ESRD without renal transplantation. To date, guidelines by the US Veterans’ Affairs and Canadian Public Health Agency are the only two available ones to advocate vaccination even, among chronic kidney disease, while most of the published guidelines advocate vaccination among ESRD. Studies on varicella among CKD patients (before progressing into ESRD) may help to give insight whether vaccinating patients once they are diagnosed with CKD of certain stages (before their progression to ESRD) may prevent this vulnerable group of patients from contracting varicella.

There is some heterogeneity in the reports of prevalence of varicella immunity among patients in ESRD. Three described the prevalence among ESRD patients who yet to contract varicella [16–18]; while four described the prevalence in already infected ESRD patients [9, 13–15]. Despite the comprehensive search, the number of available studies in the literature is low, they were summarised together in Table 2.

Another limitation is the design of the selected articles. As varicella in adult patients with ESRD has not been widely studied, there are no large-scale observational studies to date to give an impactful insight on the burden of the disease in this group of population. Most available studies are case reports and retrospective data collection and therefore are prone to selective bias (reporting bias).

Finally, future research on the cost-effectiveness on vaccinating all patients with ESRD compared to screening patients with ESRD for seronegativity before vaccinating them and monitoring will be helpful to guide national guidelines on varicella vaccination in adult patients with ESRD. This can be challenging and varies between countries depending on the robustness of national healthcare surveillance data on patients with ESRD and cost of delivering and administrating vaccines and serological tests.

## Conclusion

Varicella is a disease with great morbidity and mortality in adult patients with ESRD. Preventing varicella infection in ESRD patients is critical, and has been proven safe and reasonably efficacious in ESRD and chronic kidney disease patients.

## Abbreviations

Ab: Antibody
AUC: Area under curve
CINAHL: Cumulative Index to Nursing and Allied Health Literature
CKD: Chronic kidney disease
CMV: Cytomegalovirus
DIC: Disseminated intravascular coagulation
ESRD: End stage renal disease
Ig G: Immunoglobulin G
Ig M: Immunoglobulin M
LOS: Length of stay
MeSH: Medical subject heading
NA: not applicable
RRT: Renal replacement therapy
SORT: Strength of recommendation taxonomy
VZV: Varicella zoster virus
y.o.: years old
